# Deaf homesigners can create the foundations of phonetics and phonology without an adult linguistic model

**DOI:** 10.1016/j.cognition.2025.106233

**Published:** 2025-07-07

**Authors:** Sotaro Kita, Diane Brentari, Susan Goldin-Meadow

**Affiliations:** a Department of Psychology, University of Warwick, United Kingdom; b Department of Linguistics, University of Chicago, United States of America; c Departments of Psychology and Comparative Human Development, University of Chicago, United States of America

**Keywords:** Gesture, Sign language, Phonology, Phonetics, Homesign, Handshape discreteness of form, Duality of patterning

## Abstract

Children who are exposed to minimal linguistic input can nevertheless introduce linguistic features into their communication systems at the level of morphology, syntax, and semantics (Goldin-Meadow, 2003a). However, it is not clear whether they can do so at the level of phonetics and phonology. This study asks whether congenitally deaf children, unable to learn spoken language and living in a hearing family without exposure to sign language, introduce phonology and phonetics into the gestural communication systems they create, called *homesigns*. We focused on two foundational properties of phonetics and phonology––*discreteness of forms*, which is defined independently of meaning and thus forms the basis of *duality of patterning*. We examined index finger and open flat handshapes in deaf children’s homesigns and their hearing mothers’ co-speech gestures. We found that handshapes in deictic gestures were more discrete in homesign than in co-speech gesture. Moreover, the degree of discreteness depended on meaning (emblems vs. deictics) in co-speech gesture, but not in homesign. Children can thus create discrete forms that are meaning-independent in their homesign systems even without a model for this feature. This finding helps explain why this feature of language is universal in spoken and signed languages.

## 1. Introduction

Language development is sensitive to linguistic input. However, some features of language are developmentally *resilient* in that children can develop them even if their input provides no information or highly degraded information about them (Goldin-Meadow, 2003a). Developmentally resilient features help us understand why some linguistic features are widely found across languages. If certain linguistic properties spontaneously emerge in children’s communication systems, these features may accumulate over generations and become stable in many languages. Here, we investigated whether two foundations of phonetics and phonology are developmentally resilient.

Developmentally resilient features of language can be observed when congenitally and profoundly deaf children grow up in hearing families, without exposure to sign language. A deaf child in this situation develops a gestural communication system, called *homesign,* to communicate with family members. Homesign shows some, but not all, of the properties of language (Goldin-Meadow, 2003a). These properties are not found in the speech-accompanying gestures that the homesigner’s hearing family members produce (Flaherty, Hunsicker, & Goldin-Meadow, 2021; Goldin-Meadow and Feldman, 1977; Goldin-Meadow & Mylander, 1983, 1984, 1998). If homesigners have an opportunity to come together and form a community, they use their gestures to communicate with each another. If those gestures are then transmitted to new generations of deaf individuals, a full-blown language eventually emerges (e.g., Nicaraguan Sign Language, NSL, Kegl, Senghas, & Coppola, 1999; Senghas & Coppola, 2001; Senghas, Kita, & Özyürek, 2004; Al-Sayyid Bedouin Sign, ABSL, Sandler, Meir, Padden, & Aronoff, 2005).

The fact that some aspects of syntax and morphology can be found in homesign suggests that these properties are resilient features of language; for example, systematic word orders (Goldin-Meadow & Mylander, 1998), and morphological features such as compositional meanings derived from combinations of meaningful elements, *morphemes* (Goldin-Meadow, Mylander, & Franklin, 2007). The fact that these properties also appear in ABSL (Sandler et al., 2005) and NSL (Senghas & Coppola, 2001) points to their importance in language emergence.

In contrast to morphology and syntax, little is known about the resilient features of phonology and phonetics. Even though some work has proposed properties of emerging phonology (Brentari et al., 2021; Brentari, Coppola, Mazzoni, & Goldin-Meadow, 2012), finding these features is difficult if the emerging language does not yet have a full-blown phonology (Sandler, Aronoff, Meir, & Padden, 2011). For example, ABSL’s lexicon has very few minimal pairs (two words with distinct meanings whose forms differ by only one phonemic feature) and there is great variation in the forms of some lexical items.

We ask whether two principles that are fundamental to phonetics and phonology are found in child homesign and are thus resilient features of language. (1) The first principle is that the form of a linguistic element is discrete and categorical, not continuous. Hockett (1960) lists *discreteness* as a “design feature” of language. For example, the phonemes /p/ and /b/ differ in voice onset time (VOT, the asynchrony between opening of the lips and voicing). VOT is a physically continuous variable, but language imposes two discrete categories (voiced vs. voiceless) on this continuous dimension. (2) The second principle is that discrete forms are defined independently of the meaning of the word in which the forms appear. For example, the VOT boundary between voiced and voiceless consonants is the same across words (Lisker & Abramson, 1967), implying that the boundary is defined independently of meaning. This second principle can be seen as a manifestation of another of Hockett’s design features of language, *duality of patterning*––form and meaning are constrained by two separate sets of rules. The independence of form and meaning is important as it establishes a basis for arbitrary form-meaning mapping common in words in established languages (de Saussure, 1916/1983; Hockett, 1960).

In conventional sign languages, handshapes are phonemic and, in this sense, comparable to phonemes in spoken languages. They also display independent and hierarchically organized structure (Sandler, 1989; Brentari, 1998), and are perceived categorically (Baker, Idsardi, Golinkoff, & Petitto, 2005; Baker, Michnick-Golinkoff, & Petitto, 2006; Emmorey, McCullough, & Brentari, 2003). Two important sets of features that distinguish distinct handshapes in sign language are *selected finger* features (indicating the fingers *selected* in the handshape) and *joint* features (indicating the finger *joints* that are bent; Sandler, 1989; Brentari, 1998). In established sign languages, such as American Sign Language (ASL), these classes of features have different distributions in classifier constructions (predicates that are productively constructed from sets of meaningful components)––selected finger features are more complex when they represent an object than when they represent a hand that handles an object; joint features display the reverse pattern and are more complex when they represent a hand that handles an object than when they represent an object. Crucially, this pattern is seen not only in established sign languages (ASL, Shanghai Sign Language, Italian Sign Language), but also in an emerging sign language (NSL) in adults and children (Brentari, Coppola, Cho, & Senghas, 2017), and in homesign, the earliest stage of emerging sign language, in adults (Brentari et al., 2012) and a child (Coppola & Brentari, 2014). Importantly, this distributional pattern is *not* found in hearing individuals who know no sign language and are asked to create gestures on-the-spot, called *silent gestures*––silent gesturers use more handshape complexity (in both selected fingers and joint features) when handshapes stand for a hand that handles an object than when they stand for an object.

**Fig. 1.**
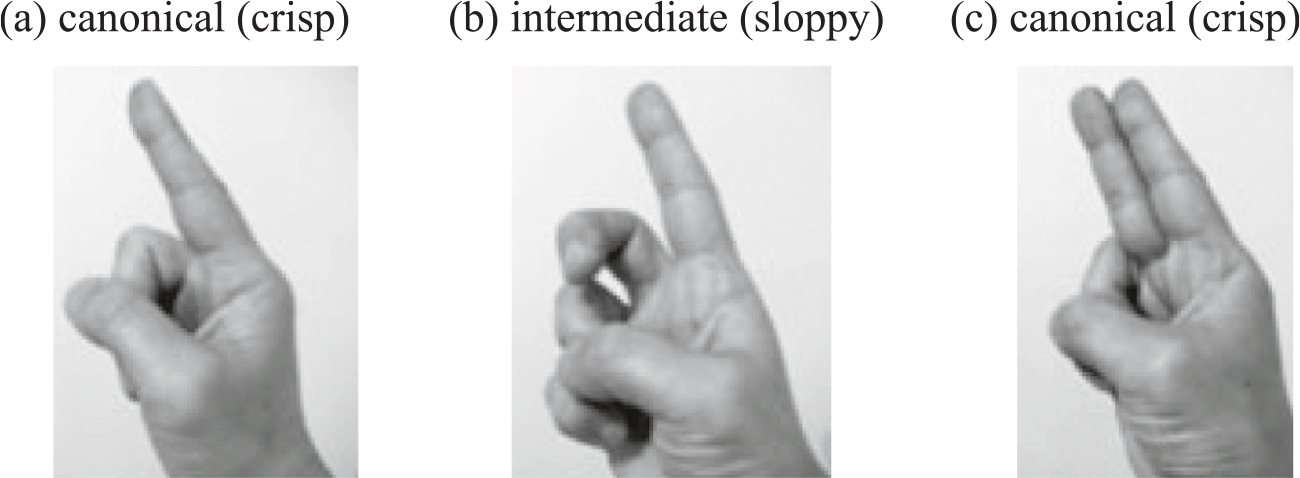
Examples of canonical (*crisp*) handshapes (a, c), in which fingers are clearly selected or unselected, and an intermediate (*sloppy*) handshape (b), in which some fingers (in this example the middle finger) are somewhere between selected and unselected. (a) canonical (crisp) (b) intermediate (sloppy) (c) canonical (crisp)

The current study explores handshape in emerging phonology, but from a different perspective. We focus on the distinction between fingers that are selected and fingers that are not selected. This distinction is found in all sign languages (Brentari, 1998, 2019) and is acquired early (Boyes Braem, 1990; Marentette & Mayberry, 2000). Fig. 1a shows a handshape where only the index figure is selected, and contrasts with Fig. 1c, where both the index finger and middle finger are selected. Handshapes that are cross-linguistically common have clear distinctions between selected and non-selected fingers (as in Fig. 1a and c, Eccarius & Brentari, 2008); all the work on handshape inventories and distribution of handshape features crucially depends on signers being able to produce and perceive this distinction. We call *canonical* handshapes (such as those in Fig. 1a,c) *crisp* handshapes. In contrast, we call *intermediate* handshapes where some fingers are in ambiguous positions (as in Fig. 1b, where the middle finger is somewhere between the selected and unselected fingers) *sloppy* handshapes.

The difference between crisp vs. sloppy handshapes distinguishes handshapes in sign language from those in co-speech gestures. Kita, van Gijn, & van der Hulst (2014) asked signers of Sign Language of the Netherlands and speakers of Dutch to describe the same animated cartoon in their respective languages. They analysed handshape in signers’ signs and in speakers’ co-speech representational gestures (which included *iconic*, *deictic,* and *metaphoric* gestures, McNeill, 1992). The signs were more likely to have crisp handshapes (e.g., Fig. 1a,c) than than the co-speech gestures, which contained many sloppy handshapes (e.g., Fig. 1b).

Here, we asked whether the distinction between selected and non-selected fingers is a resilient property of language. We observed spontaneous interactions between homesigners and their hearing mothers, and compared the handshapes produced by homesigners to the handshapes produced by the hearing mothers in their co-speech gestures.

In co-speech iconic and metaphoric representational gestures, form and meaning are linked by similarity or spatial contiguity; handshapes are thus likely to vary continuously and not be tied to canonical forms. The current research investigated handshape for deictic gestures. If a speaker points at a toy, which fingers are extended is not important to the goal of indicating the object. There is consequently no reason to avoid handshapes like the one in Fig. 1b. We therefore expect the homesigners’ hearing mothers to use sloppy handshapes (as well as crisp handshapes) in their deictic gestures.

But speakers also produce a second type of gesture––*emblems,* whose forms and meanings are fixed by convention. For example, moving the hand side to side with the palm facing the addressee and the extended index finger up means “don’t do it” in the USA. Emblems can be produced with or without speech and, unlike co-speech gestures, when produced without speech, are easily understood. Because emblems are conventional gestures with an expected form, the handshapes used in emblems should be crisp, not unlike signs in a sign language. If one is producing the *don’t-do-it* emblem, only the index finger should be extended (Fig. 1a); the middle finger should *not* be extended (Fig. 1c). Consequently, the speaker/gesturer should avoid ambiguous handshapes like Fig. 1b. We should therefore expect hearing mothers to use crisp handshapes in their emblems more often than in their deictic gestures.

As a result, whether a hearing speaker’s handshapes are discrete and crisp should depend on the meaning (function) of those gestures––handshapes in emblems, which have conventional meanings, should be more crisp than handshapes in deictic gestures, which have meanings that vary with the context of speech. In contrast, if discreteness of form is meaning-independent, as we hypothesize for homesign, homesign handshapes should *not* vary as a function of meaning––handshapes in emblems (e.g., the *don’t-do-it* gesture) and deictic gestures should be equally crisp.

To summarize, we assessed whether Hockett’s two principles––*discreteness* (linguistic form elements are discrete and categorical) and *duality of patterning* (form elements are defined independently of the meaning elements of the word in which the form elements appear)––can be considered resilient properties of language.

To explore discreteness, we coded whether handshapes were crisp or sloppy. Crisp handshapes were operationalized as those on a list of handshapes common in established sign languages. This method successfully distinguished handshapes in sign language from handshapes in co-speech gesture in Kita et al., (2014). Although Kita and colleagues used the HamNoSys list of handshapes list (Prillwitz, Leven, Zienert, Thomas, & Henning, 1989), we used Eccarius and Brentari’s (2008) list of handshapes because it is more theoretically motivated and more detailed than the HamNoSys list.

To explore duality patterning, we classified gestures as emblems or deictic gestures in homesigners and their hearing mothers, and we assessed crispness for these two categories of gestures. We predicted that crispness should vary as a function of gesture category for the hearing mothers (handshapes in emblems should be more crisp than handshapes in deictic gestures) but not for the homesigners (handshapes in emblems and deictic gestures should be equally crisp). Furthermore, homesigner’s deictic gestures should have more crisp handshapes than mothers’ deictic gestures.

## 2. Methods

### 2.1. Participants

We studied four deaf children and their hearing mothers, who lived in the US and whose data have been previously described (Goldin-Meadow & Mylander, 1983, 1984, 1998). The children had 70 to 90 dB hearing loss in both ears and attended oral-education schools for the deaf. At the point of recording, none had acquired spoken language beyond an occasional isolated word, and none was exposed to sign language. The children’s hearing parents were committed to oral education and thus communicated with their children in spoken language. However, like all hearing speakers, the parents produced spontaneous gestures when they talked (Goldin-Meadow, 2003b; McNeill, 1992). It is these gestures that were the focus of our analyses for the hearing parents.

### 2.2. Analysed recordings

The children were video recorded twice between 3 years 8 months and 4 years 11 months (each session lasted one to two hours), while interacting with a standard set of toys with their primary caregiver, the mother in all cases. These recordings were analysed in Goldin-Meadow and Mylander (1998).

### 2.3. Coding

We identified all gestures (communicative movements that did not physically move objects) produced by the children and mothers. We identified the beginning and end of the stroke phase of a gesture (Kendon, 1980; McNeill, 1992), as operationalized in Kita, van Gijn, & van der Hulst, 1998, the phase in gestural movement produced with most effort. The stroke phase typically carries the gesture’s meaning. Coding was done using a video annotation software, ELAN <https://archive.mpi.nl/tla/elan>. The coding manual can be downloaded from <https://osf.io/4x8wk/>. Frequency of gestures varied substantially across participants. We coded a maximum of 600 gestures for each child and for each mother because we knew from previous coding that, with one exception, the participants produced fewer than 600 gestures in these sessions (in other words, we capped the most prolific participant’s gestures at 600 as we were unlikely to gain statistical power by having more data from just one participant).

#### 2.3.1. Handshape form

We evaluated handshape at the beginning and end of the stroke phase of each gesture; there are thus two data points per gesture. We coded a total of 3480 data points for the four children, 2400 data points for the four mothers. Out of these data points, handshape could not be coded for 24 % of the cases (829) for children, and 20 % of the cases (476) for mothers because the hand was outside the video frame, occluded, blurred (moving too fast), holding/touching an object, or because we did not code the beginning of gestures that did not have a clear distinction between the preparation and stroke phase. This procedure resulted in 2651 data points for children and 1924 data points for mothers.

We first identified the handshape in the Eccarius and Brentari (2008) list that was closest to the observed gesture (excluding complex handshapes). We excluded complex handshapes from consideration because they are mostly specific to a particular sign language and are rare even within that sign language. We then coded whether the observed handshape was *crisp*, *sloppy*, or *relevant fingers not visible*. If finger selection was unambiguous, the handshape was coded as crisp (e.g., Fig. 1a,c). If finger selection was ambiguous, the handshape was coded as sloppy (Fig. 1b). More specifically, a handshape was considered sloppy if one of the following two statements was true: (1) The selected fingers were not all in the same position as defined by Eccarius and Brentari (e.g., all spread); this criterion was relevant only for multiple selected fingers. (2) The non-selected fingers were not all in their canonical positions (either clenched and in contact with the palm or fully extended, depending on the handshape). When we could not make the sloppy vs. crisp judgement because some fingers were not visible (e.g., due to the camera angle), handshape was coded as *relevant fingers not visible* (219 data points for children, 71 data points for mothers). These datapoints were excluded from the analysis.

In our statistical analysis, we focused on variants of the extended index finger and open flat handshapes (see Figs. A.1&2 in Supplementary Material for the canonical forms from Eccarius & Brentari). We chose this subset because a pilot study indicated that these handshapes were frequently used for the two functions that are the focus of our analysis: emblems and deictic gestures (emblems with other handshapes were quite rare––13 for children, 8 for mothers).

#### 2.3.2. Gesture meaning

Gesture meaning was classified as follows: (1) *Deictic* gestures draw attention to a particular person/object in homesign and in co-speech gesture and can be produced with either an extended index finger or an open flat hand (75 % of the data for children, 78 % for mothers). (2) *Emblems* have a defined conventional meaning in hearing gesturers, which homesigners co-opt; emblems can co-occur with speech in hearing speakers but can also be produced and interpreted without speech, e.g., *pay-attention* gesture where the index finger points upwards and the palm faces the child; a *give-me* gesture where the palm is flat and facing up (12 % of data for children, 12 % for mothers). (3) *Tracing* gestures in which the hand traces the outline of an object with the fingers (3 % for children, 1 % for mothers). (4) *Hand* gestures in which the hand represents a hand, i.e., character viewpoint gesture (McNeill, 1992) (3 % for children, less than 1 % for mothers). (5) *Shape* gestures in which the hand represents an object’s shape (1 % for children, none for mothers), (6) *Unclear* gestures in which the meaning of the gesture could not be determined (5 % for children, 4 % for mothers). Note that deictics and emblems account for most of the data for both homesigners and their mothers.

#### 2.3.3. Coding reliability

To establish intercoder reliability, a randomly selected 18 % of the data were coded by an independent coder, who was also an expert in handshape coding and was given a list of handshape options to choose from, just like the first coder. The percentage of decisions that matched between the two coders for sloppy vs. crisp was 73 % (*N* = 351) for children, 74 % (*N* = 288) for mothers. The percentage of decisions that matched between the two coders for meaning categories was 86 % (*N* = 302) for children, 86 % (*N* = 260) for mothers.

## 3. Results

Mixed effect logistic regressions were run by “glmer” with “Family = binomial” within lme4 package (version 1.1–23, Bates, Mächler, Bolker, & Walker, 2014) in R (version 4.0.0). The anonymised numerical raw data and R-markdown file can be found at <https://osf.io/4x8wk/>. The alpha level was 0.05.

**Fig. 2.**
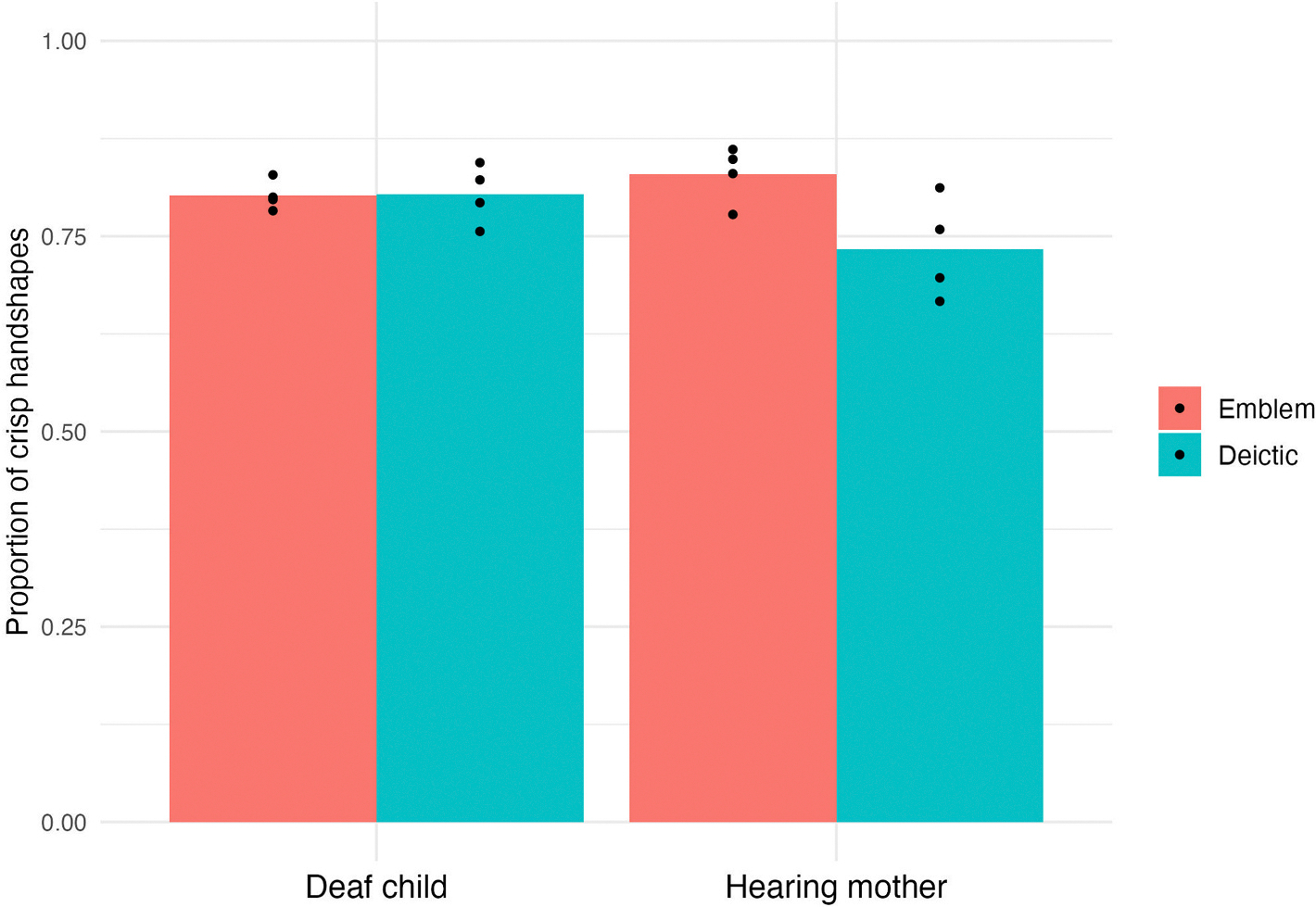
Mean proportions of gestures with crisp handshapes (handshapes in which it is clear which fingers are selected) for emblems and for deictics produced by deaf homesigners and by their hearing mothers. These gestures had either extended index finger handshapes or open flat handshapes. The higher the value, the more discrete the handshape. The black dots indicate individual participants’ data.

We investigated whether the likelihood of crisp handshapes differed as a function of meaning for the deaf homesigners and their hearing mothers. We analysed handshape for a subset of the gestures that, in form, had either extended index finger handshapes (78 % of the gestures in this subset) or open flat handshapes (22 %) (see Figures in the Supplementary Material) and, in meaning, were either a deictic (86 % of the gestures in this subset) or an emblem (14 %). This subset of gestures yielded 1403 data points for deaf homesigners and 1213 data points for their hearing mothers. We ran a mixed effect logistic regression with hand crispness (a binary variable, crisp = 1, sloppy = 0) as the dependent variable, and Age Group (child vs. mother) and Meaning (emblem vs. deictic), as independent variables (both main effects and the interaction). R’s default treatment coding was used for the fixed factors. The reference level was child for Age Group, and emblem for Meaning. We used Family as a random effect (random slopes for both main effects and the correlation, random intercepts, and their correlations). The main effects of Age Group and Meaning were not significant (Age Group, Estimate = 0.33, *p* = .24; Meaning, Estimate = 0.12, *p* = .55), but the interaction between Age Group and Meaning was significant (Estimate = −0.70, *p* = .002). See Fig. 2.

To explore the nature of the interaction, we ran four posthoc pairwise comparisons, using emmean package in R. Children’s deictic gestures had a crisp handshape significantly more often than mothers’ deictic gestures (*p* = .0027). Furthermore, mothers’ emblems had a crisp handshape more often than mothers’ deictic gestures; this comparison approached significance (*p* = .052). The interaction thus reflects the fact that handshape crispness was lower for mothers’ deictic gestures, compared to the other three conditions.

## 4. General discussions

We found that handshapes for deictic gestures were more crisp in homesigns than in mothers’ co-speech gesture (blue bars in Fig. 2). Furthermore, handshapes were equally crisp in deictics and emblems in homesigns (two bars on left in Fig. 2), but handshapes were more crisp in emblems than deictics in mothers’ co-speech gestures (two bars on right). In other words, discreteness of handshape did *not* depend on meaning in homesign, but did in co-speech gesture. Taken together, our results suggest that deaf children who are developing their homesign systems without usable linguistic input can introduce meaning-independent discreteness of forms into their communications system. Meaning-independent discreteness of forms can therefore be considered a resilient property of language.

This work contributes in two ways to the study of emerging systems, and to phonology more generally. First, it demonstrates that in order to determine whether phonology exists in a language it is crucial to observe how the entire system works; it is not sufficient to look at individual handshapes. Second, it offers an innovative way to determine whether phonology is present when minimal pairs are not yet in evidence. For several reasons, minimal pairs are not abundant in sign languages (van der Hulst & van der Kooij, 2006), and even less so in emerging languages. Brentari et al. (2012, 2021) have proposed other means of establishing a phonological system when minimal pairs are absent—*symmetry* and *productivity*. The current analysis demonstrates a third criterion for establishing a phonological system in the absence of minimal pairs—discreteness.

The mothers’ handshapes in their emblems were as discrete as the homesigners’ handshapes. Emblems are conventionalized gestures, not unlike words in language, with conventionalized forms and meanings. It is therefore not surprising that the hearing mothers used discrete forms in these gestures. This finding dovetails with the finding that emblems are processed in Broca’s area, as are words (Andric et al., 2013; Xu, Gannon, Emmorey, Smith, & Braun, 2009). It is also not surprising that the homesigners used discrete forms in their emblems because homesigners produce the emblems that their hearing parents produce (e.g., the proportion of a homesigner’s emblems that could be traced to that homesigner’s mother was 0.88 for four Chinese homesigners and 0.73 for four USA homesigners, Wang, Mylander, & Goldin-Meadow, 1993).

What is surprising is that the homesigners extend this crispness to their deictic gestures, presumably because their gestures form a single system (homesigners integrate emblems into their gesture sentences and the emblems follow the linguistic patterns of their systems). Their hearing mothers did not. This finding has two implications: (1) the homesigners are more likely to use discrete handshapes than their hearing mothers; (2) the homesigners use discrete forms whether or not the forms are emblems; the hearing gesturers use discrete forms only for emblems. Meaning thus has an impact on handshape discreteness for hearing gesturers, but not for homesigners.

Our study goes beyond previous research on the resilient features of language in important ways. Previous studies have provided evidence for developmentally resilient features in morphology, syntax, and semantics (Goldin-Meadow et al., 2007; Goldin-Meadow & Mylander, 1998; Sandler et al., 2005). Our work adds another dimension to the evidence for resilient properties of language, extending the findings to phonology and phonetics. Although phonology is slow to develop in some emerging sign languages (ABSL, Sandler et al., 2011; CTSL Brentari et al., 2021), the homesigners in our study were able to lay the foundations for a key aspect of phonetics and phonology.

Our findings provide a possible explanation for why meaning-independent discreteness of forms is a universal design feature of language. The languages of the world need to be able to be acquired by children in order to continue. If children tend to imbue their communication systems with meaning-independent discreteness of forms, this tendency should gradually accumulate over generations (Kirby, Cornish, & Smith, 2008) and eventually become established in all languages.

The meaning-independent discreteness of forms has further implications for the architecture of language. It enables arbitrary form-meaning mapping in words (de Saussure, 1916/1983; Hockett, 1960), which, in turn, facilitates a large lexicon with fine grained meaning distinctions (Ladd, 2014; Monaghan, Mattock, & Walker, 2012). Our findings thus contribute to, and build on, literature suggesting that children shape language in a significant way (Clay, Pople, Hood, & Kita, 2014).

## Supplementary Material

1

## Data Availability

The data is available at <https://osf.io/4x8wk/>.

## Appendix A. Supplementary data

Supplementary data to this article can be found online at https://doi.org/10.1016/j.cognition.2025.106233.
