# Supplementary material for: Deaf homesigners can create the foundations of phonetics and phonology without an adult linguistic model
Source: Cognition. Author manuscript; Available in PMC 2026 May 27. (PMC13215104; doi:10.1016/j.cognition.2025.106233)
Supplement: 1 [file NIHMS2169348-supplement-1.docx]

Supplementary material for “Deaf children without adult linguistic model

create foundations of phonetics and phonology”

The following figures lists the handshapes analysed in the meaning analysis.


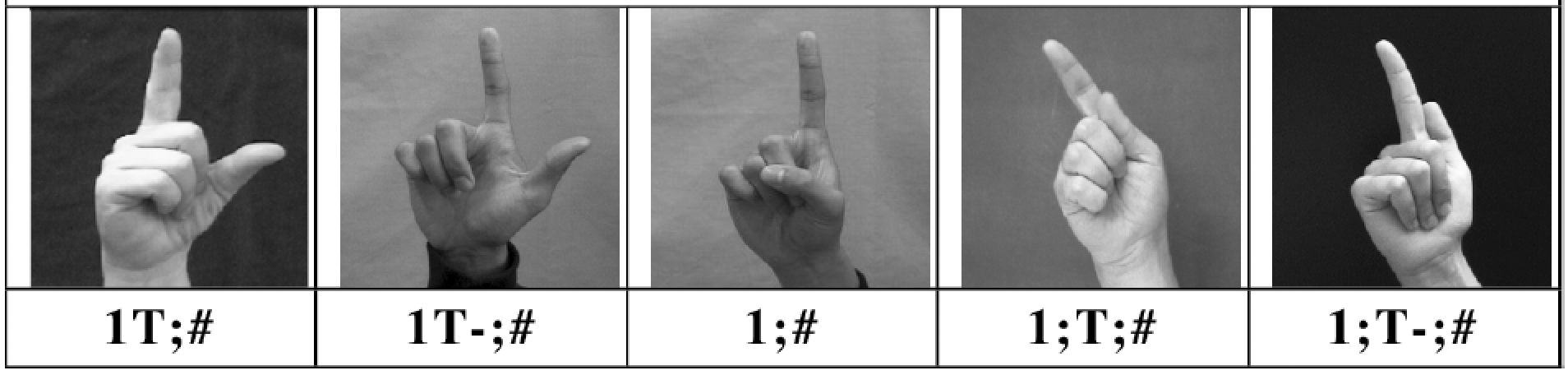


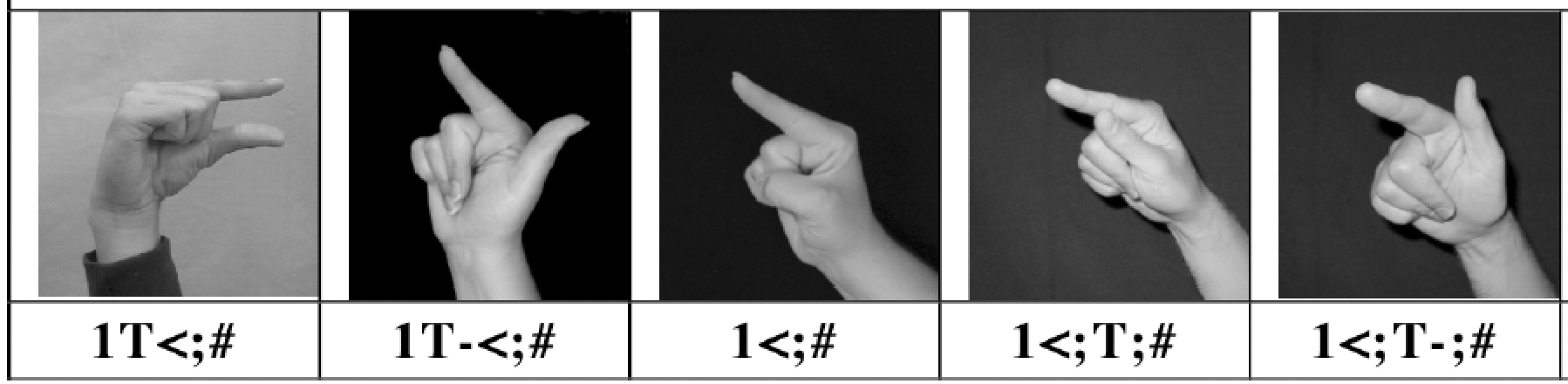


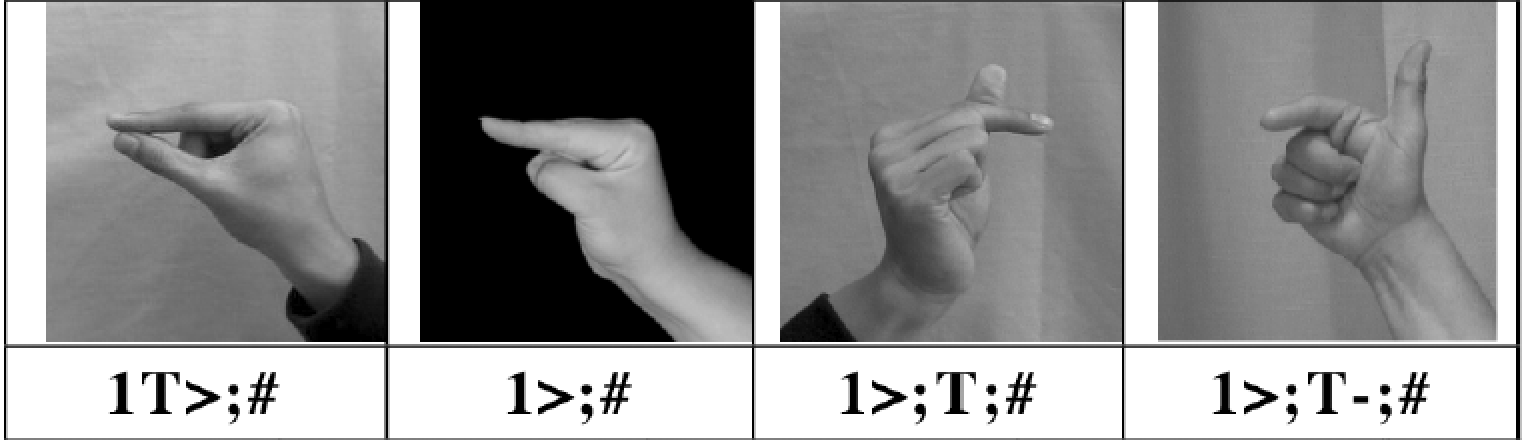


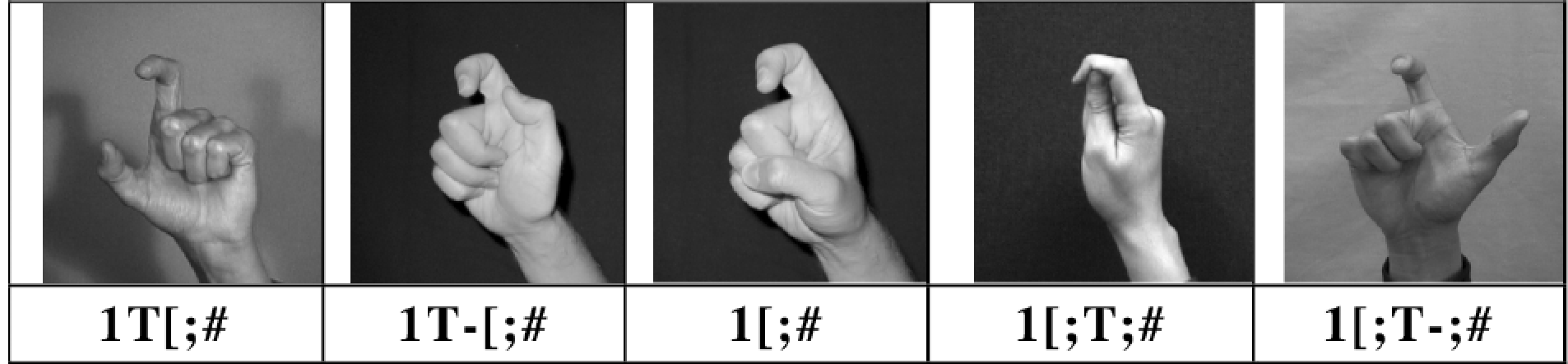


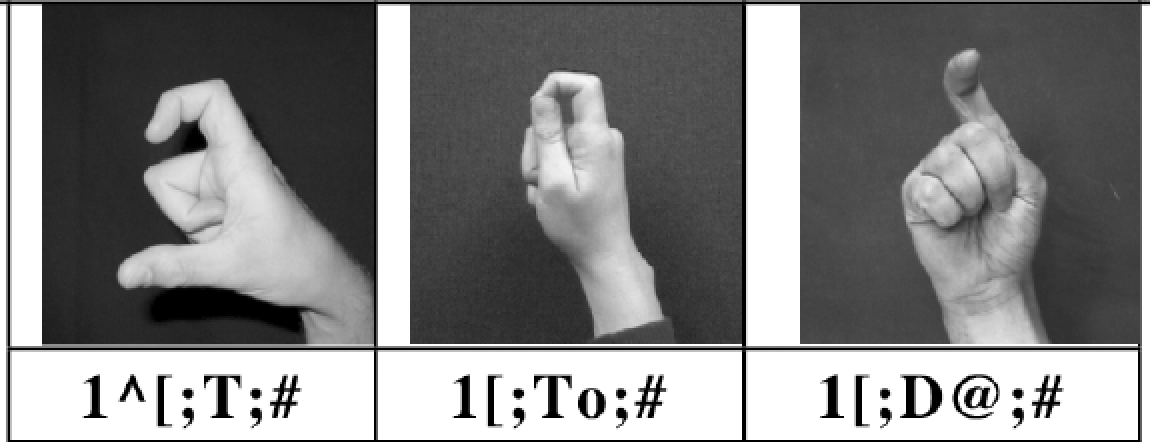


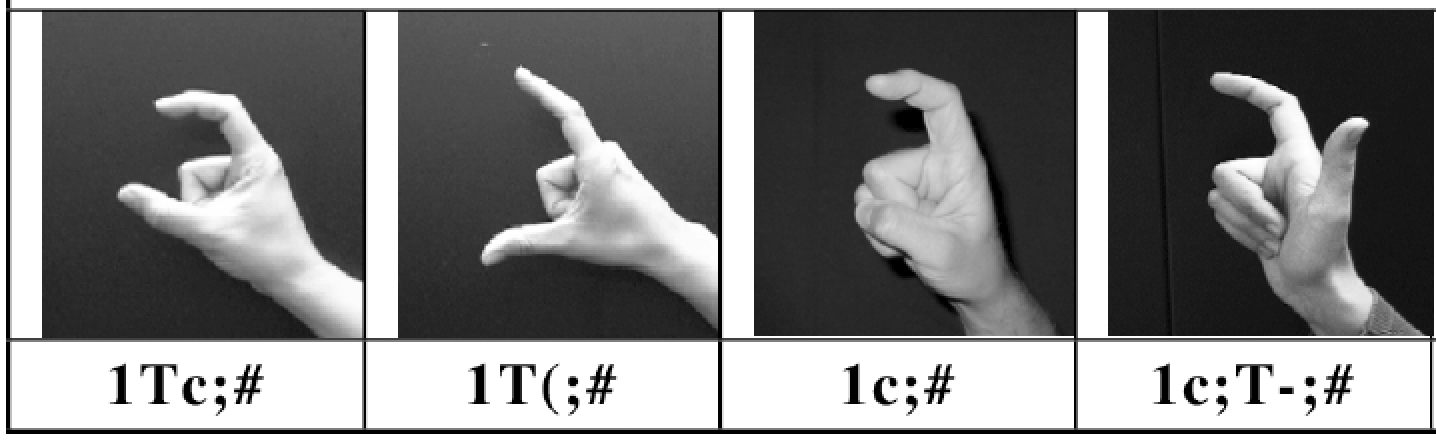


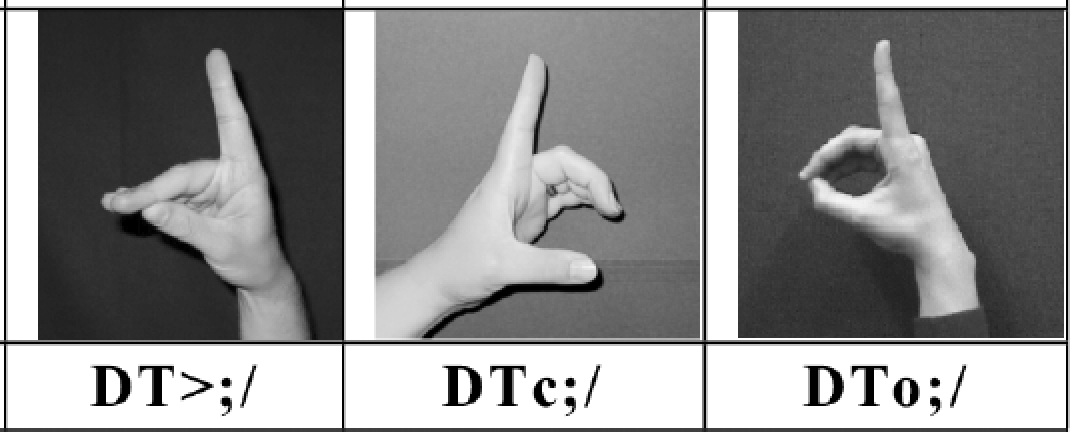


Figure A.1. Extended index finger handshapes included in the meaning analysis (Figure 3, Table 2). These are canonical handshapes, as defined in Eccarius and Brentari, (2008). Eccarius and Brentari classified them as follows: One Finger handshape – Index Finger - Extended (1^st^ row), One Finger handshape – Index Finger - Flat Open (2^nd^ row), One Finger handshape – Index Finger - Flat Closed (3^rd^ row), Bent (4^th^ and 5^th^ rows), One Finger handshape – Index Finger - Curved Open (6^th^ row), Three Finger handshape, Ring Middle and Pinky Fingers (7^th^ row). (Adapted from the Appendix of Eccarius & Brentari, 2008).


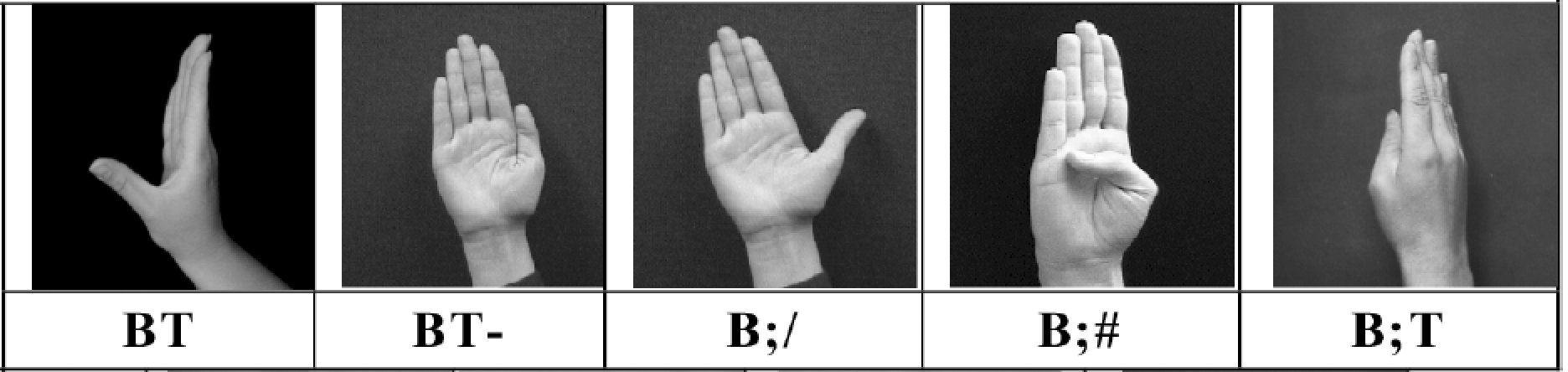


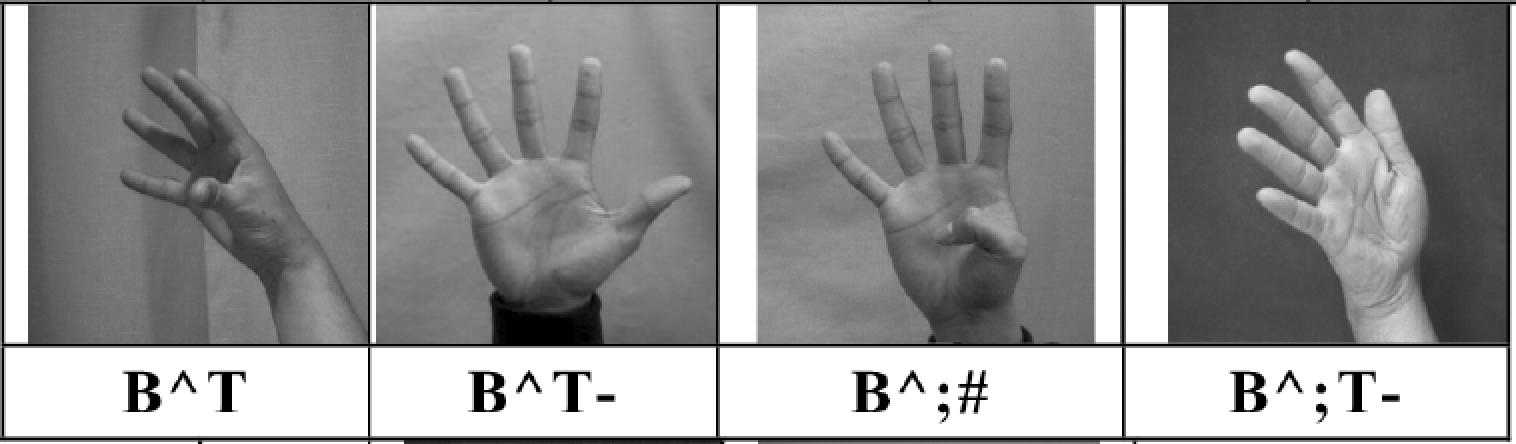


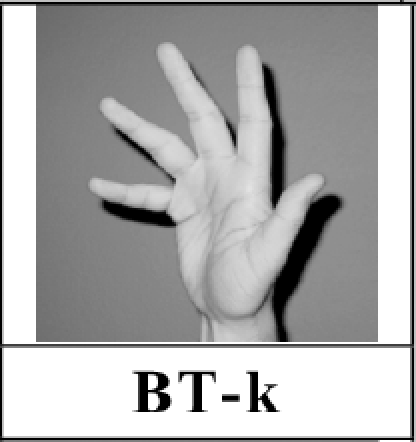


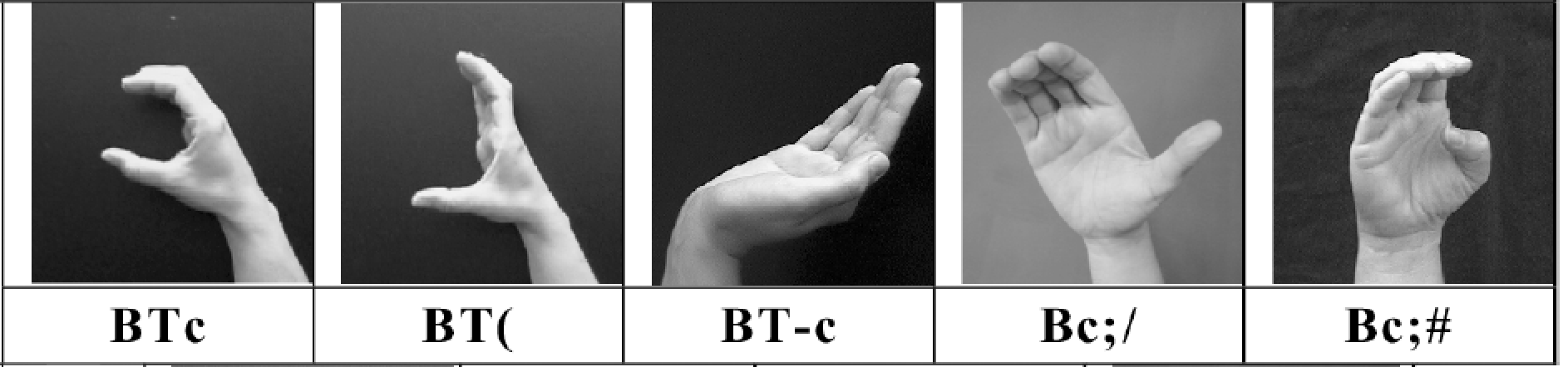


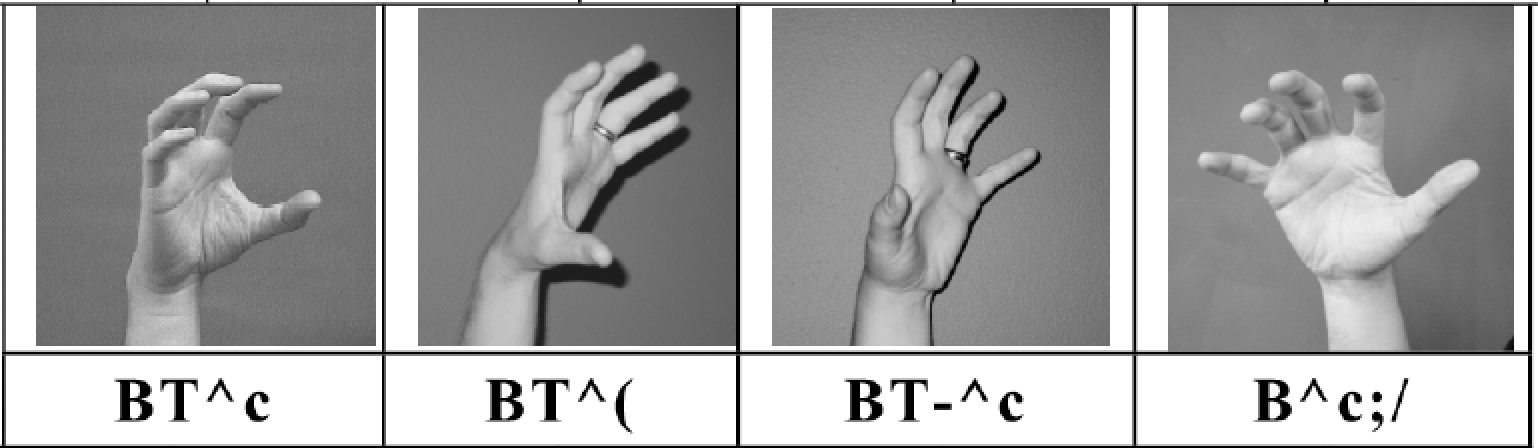


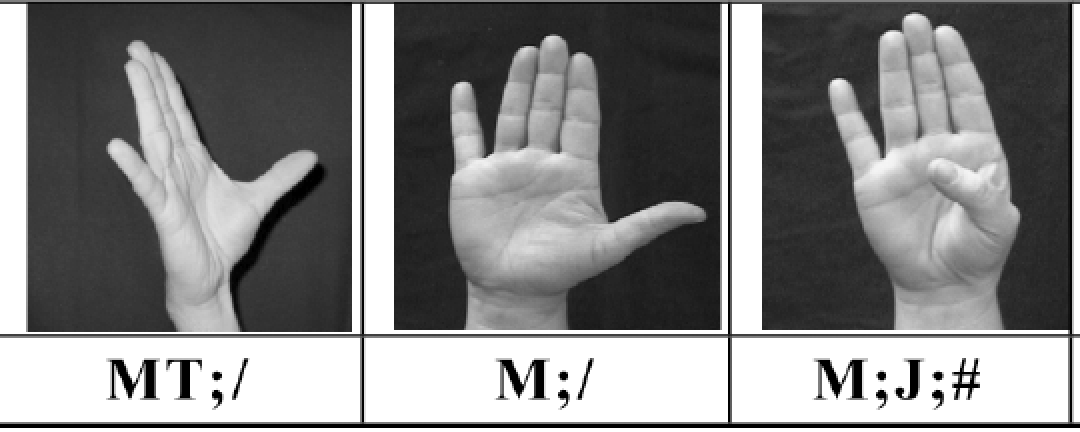


Figure A.1. Open flat handshapes included in the meaning analysis (Figure 3, Table 2). These are canonical handshapes, as defined in Eccarius and Brentari, (2008). Eccarius and Brentari classified them as follows: Four Finger handshapes – Extended - Spread (1^st^ row), Four Finger handshapes – Extended - Unspread (2^nd^ row), Four Finger handshapes – Extended – Stacked (3^rd^ row), Four Finger handshapes – Curved Open – Unspread (4^th^ row), Four Finger handshapes – Curved Open – Spread (5^th^ row), Three Finger handshapes – Index, Middle and Ring Fingers – Extended (6^th^ row) (Adapted from the Appendix of Eccarius & Brentari, 2008).
